# Flowers, Ferns, and Ward Decorations

**Published:** 1887-01-29

**Authors:** 


### Flowers, Ferns, and Ward Decorations.

[Continued from page 239.—Communications for this column should be addressed "Gardener," care of the Editor of The Hospital.]

An Appeal to those in the Sunny South.

"An ounce of practice is worth a pound of precept," and those good people who discover shortcomings in and about our hospitals with regard to this decoration, are in duty bound to suggest some means of improvement. The absence of decorations in many of these abodes makes itself very apparent to our observation ; and this is caused chiefly by the great irregularity with which flowers and plants are supplied from without. It has often occurred to me that one way of mending matters, is that those who are willing to pay and have no gardens and green-houses whence to draw perpetual contributions should commission some well-known nurseryman or gardener to dispatch weekly a bouquet of flowers, ferns, and other greenery, at an organised cost, to any one of the institutions where our sick poor are housed. This might be done for very little outlay and done systematically and carefully without trouble or inconvenience to the giver. Mr. Ware, of the Hale Farm Nurseries, Tottenham, possesses an unrivalled collection of herbaceous plants, and out of his abundance of good things would, I should say, gladly supply any demand made upon him for the purpose ; he would also pack the flowers in such a manner as passeth some men's understanding. The same may be said of Cannell and Co. of Swanby, whose well-known "Home for Flowers" might safely be taxed to provide everything the eye and nose of man could desire. All over England, there must be nurserymen ready and willing to provide the local Hospitals with any quantity of cut blossoms the whole year round. I see no reason either why the flower growers of the Continent should not be instructed to perform the same joy-giving offices, since time and season seem to make little or no difference in the floricultural wealth of Italy and southern France—if there is any difference it lies in variety and not quantity—summer and winter alike, roses bloom and violets scent the air of these well favoured regions. Why, therefore, should some of them not find their way to the bedsides of our suffering multitudes, lying weary and ill at ease within reach of the penetrating joys of "Merrie England" with the gas glaring at mid-day, while outside one's *bête noir* is indistinguishable from the apple of one's eye at two yards distance from one's own flaming nose. Men and women who can afford to fly before winter's approach as from a pestilence, into the gleaming sunshine of odorous (?) Italy, to live their days under the radiance of Mediterranean skies, could also afford to devote some of their substance to the relief of that darkness they leave behind them, and could so easily do that in the way I have mentioned, were it now suggested to them that the thing could be really accomplished. I have myself received by post from Florence, in the gloomiest days of January, gifts of exquisite, many-coloured anemones, mimosa, and jonquils obtained, no doubt at very little cost and trouble, needing only on arrival to be put, stalk downwards, in a vase of warm water, to revive them entirely, and bring out all their brilliant excellence. They have thriven in water for more than a week, none the worse for journeying by land and sea.

Guernsey, where at Christmas time, threehalfpence or twopence will procure a bunch of gloire-de-elgéus rosebuds, camus-daisies, sweet-scented cytisus (green house flowers, these, at this season in Great Britain) as well as many other blossoms, affords a firm field for the development of my plan. The boats coming regularly from thence every day, and the train service on this side of the Channel, provide every convenience for conveyance, from this land of unimpeachable pedigrees and no railways ! Here the camellias, red, white and pink, out of doors in February and March, actually hide with bloom the presence of their glossy green-leaves, growing as perfectly in the warmth of southern breezes as do those of their families coaxed into flower by dint of fire heat and unlimited persuasion in the conservatories of neighbour England, when at this season of the year they are worth their weight in gold, according to the trading London Florists. And why should not some of these help to beautify the tables and lockers of our hospital wards from one end to the other ? It needs no words to describe how welcome such gifts would be to patients and nurses alike ! in the glooming depth of wintry days.

Difficulties of Covent Garden.

There is no doubt that in summer, and, indeed, any part of the year, Covent Garden is the best and cheapest market for flowers in London ; but one must be content to rise with the lark and set out with the purveyor of milk in order to reach the scene of action in time for the early worms ! Whoso does not object to be hustled and jostled, and accosted in language of other then Belgravian refinement, and of which Billingsgate is erroneously accredited with the monopoly, may achieve most satisfactory purchases in the plant and flower line, by going first thing in the morning and bidding with the dealers for a supply at wholesale prices. Otherwise the price is too costly to admit of intemperate indulgence, unless one journey into the country itself for a stock of sweet things from the fountain head. Single buds and small bouquets, lovely as they may individually be, are as drops in an ocean of vastness within any ordinary metropolitan hospital.

People, as a rule, are lavish enough in their bestowal of flowers where they discern a fitting occasion for so doing. I have seen the entire stage of a London theatre covered with magnificent offerings of every shade and variety of horticultural glory, covered so densely that they who came forward that night to take their speechless leave of a dashing public, had to thread their way in and out of this very wilderness of wreaths and rose trimmed baskets, as through a flowery thicket in the Arabian Nights ! To be sure, it was a wonderful occasion, and the recipients of so much beauty worthy of all honours, and I think the English crowd, wrought up to a pitch of indescribable enthusiasm, who cheered, and laughed, and wept on that memorable July night, with a wild mingling of pleasure and regret, have hearts and sympathies for their neighbours as well as for their idols, and would gladly share their treasure with God's afflicted poor, when they remember whose creatures they are, and whose wealth they themselves are holding in trust—in trust *only*, and *only for a time*.

